# Study of the growth and biochemical composition of 20 species of cyanobacteria cultured in cylindrical photobioreactors

**DOI:** 10.1186/s12934-023-02035-z

**Published:** 2023-02-24

**Authors:** Douglas Henrique Baracho, Ana Teresa Lombardi

**Affiliations:** 1 Programa de Pós-Graduação em Ecologia e Recursos Naturais (PPGERN), Universidade Federal de São Carlos (UFSCar), Rod. Washington Luís km 235, São Carlos, São Paulo CEP 13565-905 Brazil; 2 Departamento de Botânica (DB), Universidade Federal de São Carlos (UFSCar), Rod. Washington Luís km 235, São Carlos, São Paulo CEP 13565-905 Brazil

**Keywords:** Biotechnology, Proteins, Carbohydrates, Lipids, Pigments, Phycocyanin, Carotenoids, Polyhydroxyalkanoates, Antioxidant

## Abstract

**Background:**

Cyanobacteria are prokaryotic organisms with wide morphological and metabolic diversity. By means of photosynthesis, they convert inorganic compounds into biomolecules, which may have commercial interest. In this work, we evaluated 20 cyanobacterial strains regarding their physiological aspects such as growth, photosynthesis and biochemical composition, some of which are revealed here for the first time. The organisms were cultivated in cylindrical photobioreactors (CPBR) for 144 h and the biomass was obtained. The light inside cultures was constant throughout experimental time and maintained at the saturation irradiance (Ik) of each species. Culture pH was maintained within 7.8 and 8.4 by automatic CO_2_ bubbling. Growth rate, dry biomass, chlorophyll *a*, carotenoids, phycocyanin, proteins, carbohydrates, lipids, polyhydroxyalkanoate (PHA) and antioxidant activity were determined.

**Results:**

The proportionality of the biochemical composition varied among species, as well as the growth rates. *Leptolyngbya* sp. and *Nostoc* sp. (CCIBt3249) showed growth rates in the range of 0.7–0.8 d^−1^, followed by *Rhabdorderma* sp. (~ 0.6 d^−1^), and *Phormidium* sp. (~ 0.5 d^−1^). High carotenoid content was obtained in *Rhabdoderma* sp. (4.0 μg mL^−1^) and phycocyanin in *Leptolyngbya* sp. (60 μg mL^−1^). Higher total proteins were found in the genus *Geitlerinema* (75% DW), carbohydrates in *Microcystis navacekii* (30% DW) and lipids in *Phormidium* sp. (15% DW). Furthermore, *Aphanocapsa holsatica* showed the highest antioxidant activity (65%) and *Sphaerocavum brasiliense*, *Microcystis aeruginosa*, *Nostoc* sp. (CCIBt3249) and *A. holsatica* higher levels of PHA (~ 2% DW).

**Conclusions:**

This study reports on the biochemical composition of cyanobacteria that can impact the biotechnology of their production, highlighting potential strains with high productivity of specific biomolecules.

**Supplementary Information:**

The online version contains supplementary material available at 10.1186/s12934-023-02035-z.

## Background

In recent years, with the increase in world population and industrialization, the search for sustainable sources of raw materials for everyday products has had a growing interest [1]. Cyanobacteria can be considered as promising organisms for obtaining these materials [2, 3]. They are prokaryotes, metabolically and morphologically diverse and are present in different habitats, predominantly in aquatic environments [4]. From oxygenic photosynthesis, they are responsible for a large part of the fixation of atmospheric carbon dioxide, a greenhouse gas [5].

As photosynthetic organisms, cyanobacteria convert inorganic compounds and light energy into organic products. The absorption of light energy is directed by photosynthetic pigments, namely chlorophylls, carotenoids and phycobilins, the latter only present in cyanobacteria and red algae [1]. There are several advantages of generating biomass from these organisms and among them, the high growth rate [6] and atmospheric CO_2_ biofixation [5]. In addition, they can have their production coupled to gaseous industrial waste or cultivated in industrial effluents [7] and thus have the production costs reduced.

It is known that some cyanobacteria are a source of human and animal food due to their high protein content [1]. Carbohydrates from cyanobacteria can be used to produce bioethanol, as well as lipids to obtain biodiesel [8], and for the human diet, the omega-3 [1]. In addition, photosynthetic pigments such as chlorophyll* a*, carotenoids and phycobiliproteins have their importance for the nutraceutical and pharmaceutical industries [9]. Among the new possibilities for using cyanobacteria biomass is the production of polyhydroxyalkanoates (PHA) for the production of bioplastic, as a promise for the future [10]. However, to obtain these biomolecules, new strains need to be characterized in terms of productivity and growth [11], as well as optimized production processes aiming at higher yields [12].

In addition to the content of intrinsic biomolecules of each species, it is known that the proportions of these molecules vary in response to abiotic changes in culture medium in which they are inserted, such as, for example, the availability of nutrients. It is known that batch cultivations are simple and their internal conditions vary over time [13]. In their work Vargas et al. [14] observed that the percent of proteins doubled and of carbohydrates decreased between the adaptation and exponential phases in 12 species of cyanobacteria. The control of cultivation conditions to properly satisfy the metabolic needs in cyanobacterial production systems is reflected in the quality of the final biomass. This is particularly important for industrial production, as product quality needs to be preserved.

A determining factor for the success of microalgae production is the bioreactor used [15]. The control of the intensity of incident light, pH, aeration, CO_2_ and temperature reflects on the yield and quality of the biomass [4]. Cultures grown in natural environments and open reactors are more susceptible to contamination, temperature fluctuations and evaporation [1] than controlled systems, and tend to be more selective, due to difficult-to-control conditions such as sunlight and biological contamination. On the other hand, they are less prone to heating problems since evaporation is the natural way of heat dissipation. Therefore, a limited number of species are successful in open systems, such as *Spirulina* Turpin ex Gomont and *Dunaliella* Teodoresco [4]. The successful growth of cyanobacteria in closed bioreactors is due to adjustments of the inherent properties of the reactor to optimally accommodate each species [16]. Closed photobioreactors have advantages over open ones, as they have high biomass productivity and low probability of contamination [12].

Considering the great diversity of cyanobacteria, the objective of this work was to prospect 20 species cultivated in tubular reactors, focusing on growth under ideal conditions and evaluating biomolecules (proteins, carbohydrates, lipids, chlorophyll *a*, carotenoids, phycocyanin and polyhydroxyalkanoates), as well as antioxidant activity of methanolic extracts produced from biomass. In our study, we used a photobioreactor with internal adjustments for light, pH, CO_2_ insertion, and culture agitation via bubbling. With this, we guaranteed ideal growing conditions sustaining the production of healthy biomass until the last experimental day. As a laboratory scale system (2 L), heating was not a problem under the conditions used. Our results are a contribution to understanding the physiology and technology of production and the potential for commercial use of new species of cyanobacteria.

## Results and discussion

### Growth parameters

**Fig. 1:**
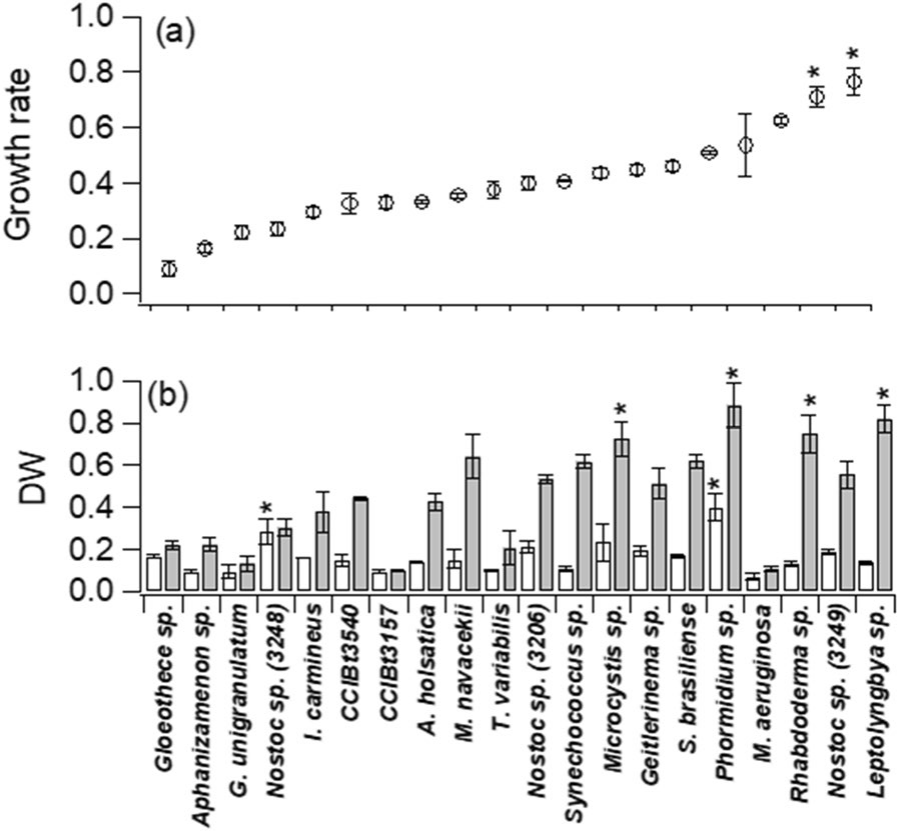
In **a** growth rate (d^−1^) and in **b** dry biomass (g L^−1^) for cyanobacterial strains as a function of experimental time. In **b** white bars represent biomass at 48 h and gray bars represent dry biomass at 144 h. Error bars represent the standard deviation from the mean (n = 3). The asterisk (*) indicates the highest value (ANOVA, p < 0.05)

Growth rate under optimal conditions is an intrinsic characteristic of the species, thus the variation obtained in this study among species can be expected. Figure 1 shows the results of population growth for the 20 species of cyanobacteria grown in the CPBR, in Fig. 1a the growth rates (µ), and Fig. 1b the dry biomass (g L^−1^) obtained in 48 h and 144 h. Growth rates ranged from 0.1 to 0.8 d^−1^, with the majority (65%) within 0.3 to 0.5 d^−1^. *Leptolyngbya* sp. (0.77 d^−1^) and *Nostoc* sp. CCIBt 3249 (0.71 d^−1^) stood out with the highest growth rates. In a study comparing the growth rates of *Chlorella vulgaris* Beijerinck with 4 species of cyanobacteria (*Aphanizomenon ovalisporum* Forti*, Anabaena planctonica* Brunnthaler*, Borzia trilocuraris* Cohn ex Gomont and *Synechocystis* sp. C.Sauvageau) Mendez et al. [17] showed a variation from 0.2 to 1.2 d^−1^, with 0.6 d^−1^ as the highest value among the cyanobacteria *Synechocystis* sp. Comparing these results with those of the present study, it is observed that *Leptolyngbya* sp. had 25% higher growth rate, the cyanobacterium with the highest growth rate among the ones we studied.

Differences in growth rates between strains of the same genus are common. In a study with 3 species belonging to the genus *Spirulina*, the highest growth rate was observed in *S. platensis* (Gomont) Geitler (1.4 d^−1^) and the lowest in *S. lonar* Turpin ex Gomont (0.63 d^−1^) [18]. The *Nostoc* species (CCIBt3206; CCIBt3249) studied here had growth rates approximately 2 × higher than *Nostoc muscorum* C. Agardh ex Bornet & Flahault also cultivated under CO_2_ supplementation [6]. While these differences may simply be due to an intrinsic characteristic of the species, they may be related to growing conditions. Our CPBR has among its advantages the regulation of light as the culture density increases, which, together with pH control by internal CO_2_ bubbling, provides good growth conditions, assuming that soluble nutrients are available in excess.

Regarding dry biomass, all species showed an increase between 48 and 144 h, as expected. At the end of the experimental period, four species showed greater accumulation of biomass (~ 0.8 g L^−1^), namely, *Leptolyngbya* sp., *Rhabdoderma* sp., *Phormidium* sp. and *Microcystis* sp. Our results showed that some species were particularly promising in accumulating biomass. This applies to *M. navacekii*, *Nostoc* sp. (3206), *Synechococcus* sp., *Microcystis* sp., and *Phormidium* sp. that presented a growth rate in the range of 0.3 to 0.5 d^−1^, but similar biomass to those that presented a growth rate of 0.6 to 0.8 d^−1^ (*Rhabdoderma* sp., *Nostoc* sp., *Leptolyngbya* sp.). The range of dry biomass at the end of cultivation for 65% of the species we investigated varied from 0.4 to 0.8 g L^−1^. Comparing our results for *Synechococcus* sp. (0.6 g L^−1^) with those of the same gender in the study by Shanmugam et al. [19], we obtained twice as much biomass, even considering that in Shanmugam et al. [19], 24 h longer culture period was performed.

With a growth rate of 0.8 d^−1^ and dry biomass of ~ 0.8 g L^−1^ in 144 h, the cyanobacterium *Leptolyngbya* sp. can be a promising organism for obtaining biomass (and its associated products). The accumulated biomass in *Leptolyngbya* sp. is twice that found for *Synechococcus* sp. Nägeli under similar growth conditions [5]. It is likely that the conditions we used favored the highest accumulation of biomass compared to the literature data for cyanobacteria. The maintenance of the pH through automatic insertion of CO_2_ favors photosynthesis, the primary process for the generation of biomass. Additionally, under our conditions light intensity was also kept constant inside the CPBR according to the densification of the culture, reducing effects of self-shading.

**Fig. 2:**
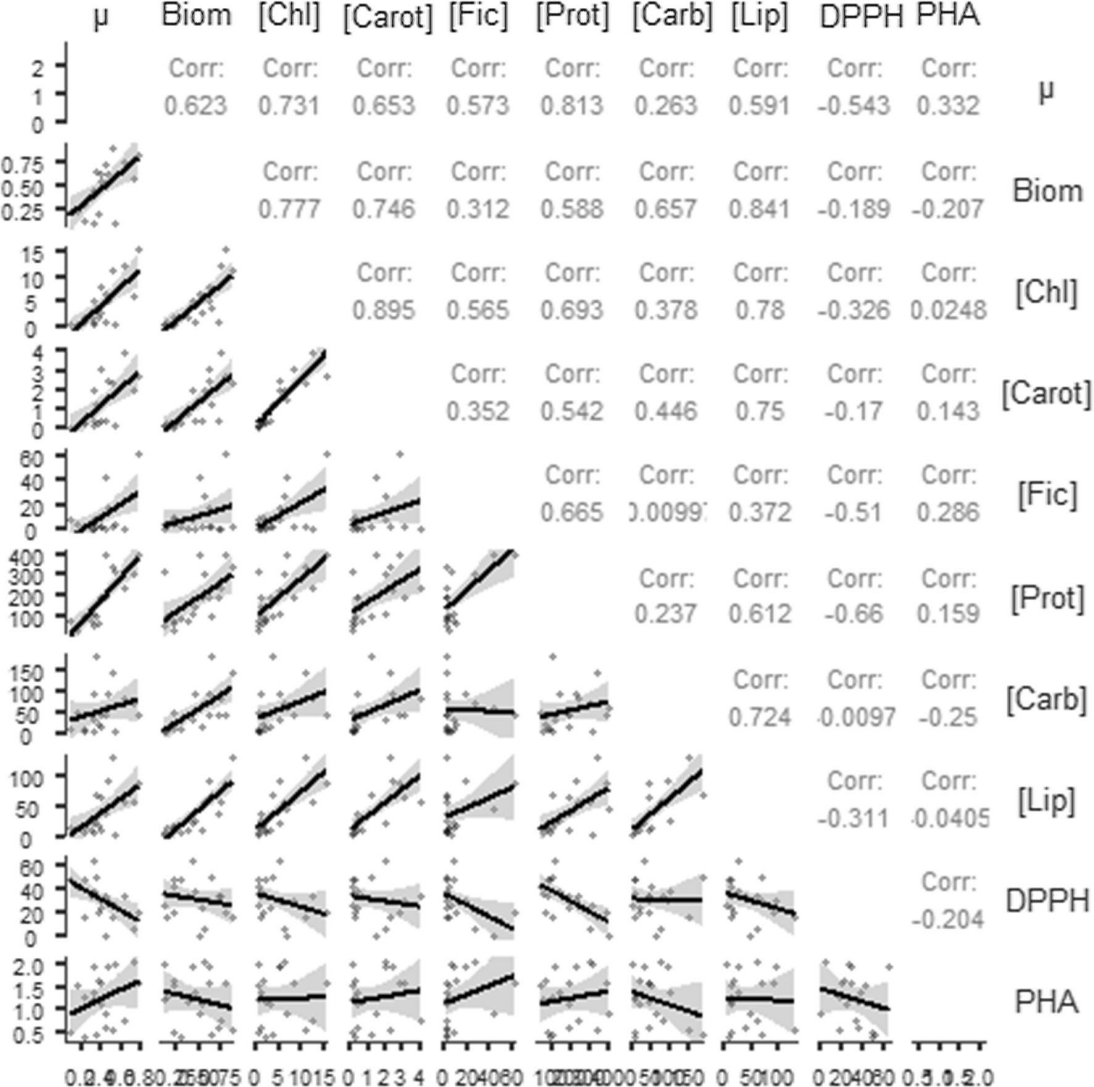
Correlation matrix for information on cultivation in the 144 h experimental time: growth rate (µ), dry biomass (Biom), antioxidant activity (DPPH) and determined total biomolecules (chlorophyll a—Chl a, Phycocyanin—Fic, Carotenoids—Carot, Carbohydrates—Carb, Proteins—Prot, Lipids—Lip, Polyhydroxyalkanoates—PHA)

Literature data show that the correlation between growth rate and biomass yield can vary [20]. In that study two species of microalgae and two cyanobacteria were studied and showed that in different concentrations of CO_2_ the highest growth rates did not correspond to higher biomass yield. We understand that two reasons can result in greater biomass at lower growth rates, either larger cells and/or greater accumulation of biomolecules per cell unit. In the present study we observed a correlation (Fig. 2) coefficient of ~ 0.62 between these parameters (growth rate *vs* biomass), considering all 20 species analyzed. However, we observed that biomass correlated better with chlorophyll *a* (~ 0.78), carotenoids (~ 0.75) and lipids (~ 0.84).

We emphasize that much of the literature performs cyanobacterial cultivations below 100 µmol of photons m^−2^ s^−1^ [3]. Here, cultivation in Ik (mostly above 100 µmol photons m^−2^ s^−1^) may have favored cell division, resulting in higher growth rates compared to the literature. In fact, the cyanobacteria *S. platensis* (Gomont) Geitler exposed to 40, 60 and 120 µmol of photons m^−2^ s^−1^ showed a higher growth rate when exposed to higher light intensity [21].

### Biochemical composition

The biochemical composition data are reported for the 144 h experimental time. Aiming on the production of biomolecules in healthy biomass, using the CPBR we guaranteed ideal cultivation conditions of light and pH until the last day of cultivation, temperature was controlled, and nutrients given in excess.

**Fig. 3:**
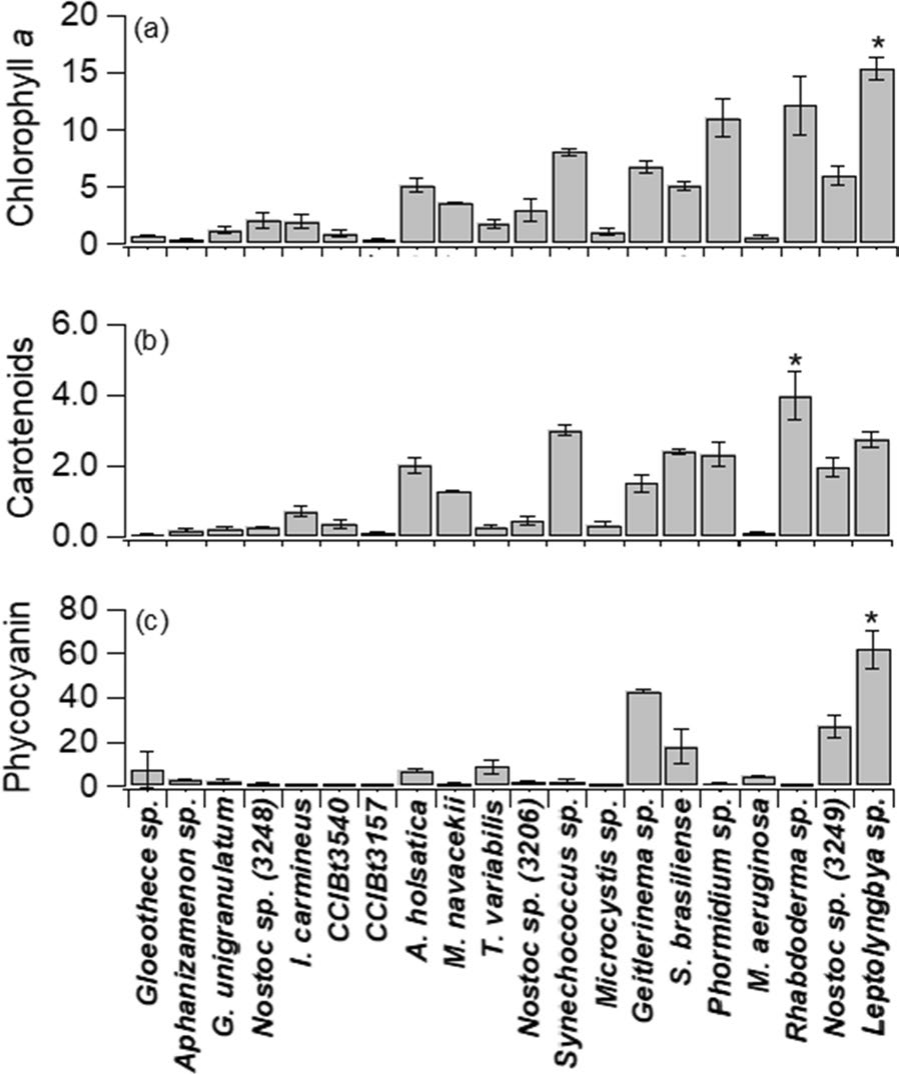
Pigment concentration in 144 h for the 20 species of cyanobacteria cultivated. In **a** chlorophyll a (µg mL^−1^); in **b** total carotenoids (µg mL^−1^); in **c** phycocyanin (µg mL^−1^). Error bars represent the standard deviation from the mean (n = 3). The asterisk (*) indicates the highest value (ANOVA, p < 0.05)

The concentrations of chlorophyll *a*, carotenoids and phycocyanin pigments at 144 h is shown in Fig. 3. As reported in the literature, different species of cyanobacteria have different proportions of pigments in their biomass [22]. The cyanobacteria grown in this study showed higher amounts of chlorophyll *a* than most published results. *Leptolyngbya* sp. produced the most and, yielding 15 µg mL^−1^ chlorophyll *a* in 6 days (2.5 µg mL^−1^ d^−1^) it is compatible with the commercialized cyanobacteria, *Spirulina platensis* [23]. *Leptolyngbya* sp. produced ~ 2.5 × higher chlorophyll *a* than that reported for the same *Spirulina* species in another study [24]. We suggest that *Leptolyngbya* sp. together with *Rhabdoderma* sp. and *Phormidium* sp., which also reached expressive values of chlorophyll *a*, are promising candidates for large-scale cultures aiming at the commercial production of this pigment. There was a positive correlation of 0.73 for chlorophyll *a vs* growth rate, in line with the literature [25]. Paliwal et al. [25] reported that a 20% reduction in growth rate resulted in a 60% reduction in chlorophyll *a* concentration in *Synechocystis* sp.

Regarding total carotenoids (Fig. 3b), after 144 h of cultivation, *Rhabdoderma* sp. stood out, reaching ~ 4 µg mL^−1^ of this pigment. Carotenoids are commercially desired photosynthetic pigments to be applied in the pharmaceutical, cosmetic and food industries [9]. As with chlorophyll *a*, the concentrations of carotenoids we obtained were generally higher than those found in the literature, with emphasis on *Rhabdoderma* sp. This species, with 0.67 µg mL^−1^ d^−1^ of carotenoids, produced about 5 × more than *Spirulina platensis* (0.13 µg mL^−1^ d^−1^) [24], widely commercialized. In a study with *Anabaena cylindrica* Lemmermann*, Anabaenopsis elenkinii* V.V.Miller*, Anabaena torulosa* Lagerheim ex Bornet & Flahault and *Nostoc* sp. Vaucher ex Bornet & Flahault, Schagerl, Müller [22] found the highest yield of total carotenoids in *Anabaenopsis elenkinii*. Compared to this species, the carotenoid yield was 13 × higher in *Rhabdoderma* sp. (this study). In addition, other viable candidates at the end of 144 h for carotenoid production (~ 2.5 µg mL^−1^) were *A. holsatica*, *Synechococcus* sp., *S. brasiliense*, *Phormidium* sp., *Nostoc* sp. (3249) and *Leptolyngbya* sp.

Phycocyanin concentrations varied among species (Fig. 3c). The highest values of phycocyanin were found in *Nostoc* sp. CCIBt3249 (27 µg mL^−1^), *Geitlerinema* sp. (43 µg mL^−1^) and *Leptolyngbya* sp. (62 µg mL^−1^) in 144 h. This pigment is of commercial interest as a natural dye. Our values for *Leptolyngbya* sp. are up to 9 × higher than that found for *Spirulina* sp., cultivated under controlled conditions in other studies [23, 26]. We emphasize that phycocyanin is just one of the pigments present in phycobilisomes [1], and species that had low phycocyanin values may have other types of phycobiliproteins in their composition, such as phycoerythrin and allophycocyanin [27]. We add that, as in our work, kept in Zarrouk culture medium and ideal cultivation conditions, the cyanobacterium *Spirulina platensis* showed higher concentrations of phycocyanin than chlorophyll *a* [23].

As expected, for all strains we investigated there was an increase in the accumulation (µg mL^−1^) of proteins, carbohydrates and lipids between 48 and 144 h (Additional file 1: Fig. S1). Considering these biomolecules, the highest concentrations were for proteins. Similarly, in a study that aimed at characterizing cyanobacteria biomass *Synechocystis* sp. C.Sauvageau and *Borzia trilocularis* presented higher levels of proteins in comparison to carbohydrates were found [17]. In the present research, total proteins (µg mL^−1^) correlated with growth rate (0.8), and the highest values of total proteins were found in species that showed higher growth rates. Other works have also reported the relationship between protein and growth rate in exponentially growing cells, as observed for *M. aeruginosa* Kützing [28]. It is known that in healthy cells and exponential growth, many of the proteins present in cyanobacteria are structural, constituents of genetic material [29], and thus participate in population increase. We also obtained a positive correlation (0.69) between proteins and chlorophyll *a*, similar to that described for *Gleiterinema lemmermannii* (Wołoszyńska) Anagnostidis [7].

Considering the content of biomolecules per unit biomass (% DW) we emphasize that in the present research we did not obtain variation in the content of these biomolecules with the advancement of experimental time. For example, the percent composition of proteins, carbohydrates and lipids in the dry biomass were similar within each biomolecule at 48 h (data not shown) and 144 h, confirming that the conditions used in this research supported a healthy cell growth.

The percent of proteins, carbohydrates and lipids in 144 h in relation to the dry biomass of each species are reported in Table 1. There was a variation between the total percentages of proteins (19–64%), carbohydrates (2.5–34%) and lipids (1.5–20%) among the cultivated species. At the end of 144 h, the two species of the genus *Geitlerinema (Geitlerinema unigranulatum* and *Geitlerinema* sp.) stood out with ~ 77% of total proteins. The highest total carbohydrates contents (% DW) were found in *M. navacekii* (28%) and *Aphanizamenon* sp. (22%). When the lipid content of the biomass was evaluated, *Phormidium* sp. and *S. brasiliense* showed higher values, ~ 15%. Our results are in line with data from the literature, which shows variation in the biochemical composition between different species of cyanobacteria [8]. Similar to our study, Vargas et al. [14] evaluated the levels of carbohydrates, lipids and proteins of 12 cyanobacteria and showed a variation of 16–37% for carbohydrates, 37–52% for proteins and 8–13% for lipids among species.

**Table 1:** Proteins, carbohydrates and lipids content in the biomass (% DW) of the investigated cyanobacteria

Species	Proteins	Carbohydrates	Lipids
*Gloeothece* sp.	35.6 (16.0)	3.5 (0.9)	6.7 (0.7)
*Aphanizamenon* sp.	10.3 (1.3)	22.2 (3.9)*	6.7 (1.9)
*G. unigranulatum*	75.8 (21.2)*	4.4 (0.5)	11.1 (2.6)
*Nostoc* sp.* CCIBt3248*	26.2 (6.4)	2.6 (0.3)	2.4 (0.9)
*I. carmineus*	19.0 (6.0)	11.6 (3.2)	3.8 (1.4)
*CCIBt 3540*	31.3 (15.5)	21.9 (5.0)*	6.2 (1.6)
*CCIBt 3157*	49.0 (2.9)	4.1 (1.2)	8.5 (1.5)
*A. holsatica*	25.5 (10.6)	7.3 (1.2)	5.6 (0.3)
*M. navacekii*	13.7 (3.8)	27.7 (2.4)*	10.5 (2.4)
*T. variabilis*	57.0 (27.8)	10.7 (2.6)	8.6 (0.7)
*Nostoc* sp.* CCIBt3206*	34.4 (5.5)	3.3 (0.3)	2.2 (0.3)
*Synechococcus* sp.	30.4 (3.4)	7.4 (1.9)	10.7 (3.2)
*Microcystis* sp.	25.5 (10.6)	5.6 (2.2)	5.6 (2.4)
*Geitlerinema* sp.	77.9 (5.7) *	11.6 (1.4)	9.2 (2.0)
*S. brasiliense*	36.0 (4.9)	14.7 (2.1)	15.2 (2.7)*
*Phormidium* sp.	38.0 (5.3)	16.7 (2.7)	14.8 (5.1) *
*M. aeruginosa*	33.3 (1.7)	1.7 (0.4)	5.7 (1.0)
*Rhabdoderma* sp.	31.8 (5.3)	11.2 (1.8)	7.7 (1.2)
*Nostoc* sp.* CCIBt3249*	54.7 (7.8)	12.5 (1.9)	10.5 (1.6)
*Leptolyngbya* sp.	50.7 (9.7)	5.0 (0.1)	11.2 (1.6)

Statistics: asterisk (*) indicates the highest value (ANOVA, p < 0.05). Numbers in parenthesis correspond to the standard deviation of the mean (n = 3)

The percent of proteins in the dry biomass we obtained are higher than those found for other cyanobacterial strains reported in the literature [14]. With 75% proteins in dry biomass, *G. unigranulatum* e *Gleiterinema* sp. produced 2.4 × more of this biomolecule than what was found for *Geitlerinema lemmermanii* in healthy conditions [7]. *G. unigranulatum* produced proteins in amounts similar to those found for commercial species under ideal cultivation conditions, such as *Synechocystis* sp. [25] and 1.2 × higher than *Anabaena variabilis* Kützing ex Bornet & Flahault [30]. The genus *Geitlerinema* has already been described as an outstanding lineage for the production of protein-rich biomass compared to other species [7], which was confirmed in this research. In a study with *Spirulina platensis* in a photoautotrophic system, the highest protein content was 55% at the end of the third day of cultivation and there was a reduction to 25% at the end of the 10th day [31], when probably some stress began. We attribute the high concentration of proteins in the strains investigated in this research to ideal cultivation conditions, since the protein content in *G. unigranulatum* and *Gleiterinema* sp. were similar at 48 h (data not shown) and at 144 h (data not shown). Thus, we confirm that the CPBR used provided growth conditions at ideal levels to maintain the health of the culture for up to 144 h.

Analyzing carbohydrates production, we found that *M. navacekii* stood out at 144 h, with its production ~ 1.4 × higher than that of *Anabaena sphaerica* Bornet & Flahault at the end of 192 h of growth in another study [26]. With 28% carbohydrates on a dry biomass basis, *M. navacekii* was similar to *Spirulina platensis* (~ 30%) under appropriate cultivation conditions [31]. Our values were also close to those found for *Spirulina maxima* (Setchell & N.L.Gardner) Geitler [15] and *Synechocystis* sp. [25]. In the present study, the concentration of total carbohydrates, unlike proteins, showed a low correlation with growth rate, but a correlation of 0.72 with total lipids. This confirms that in cyanobacteria this biomolecule is associated with the formation of glycolipids, an important molecule in the stability of membranes and, in cyanobacteria, it also participates in the functioning of heterocysts and akinetes, as shown in Garg; Maldener [32].

Lipids are reserve molecules and are part of cell membranes [33]. *Phormidium* sp. (14.8% DW) and *S. brasiliense* (15,2% DW) showed to be promising strains for lipids production, but most of the strains investigated here produced less than 10% of this biomolecule. These results agree with the literature [34, 35]. Investigating 12 strains of cyanobacteria, Vargas et al. [14] showed a variation of 8 to 13% in the lipid percent of the strains. Species with a high lipid content may be of great interest and be cultivated for biodiesel production [4]. The lipid content of *S. brasiliense* is ~ 1.2 × greater than that found for *Nodularia* sp. Mertens ex Bornet & Flahault and *Nostoc* sp. [14], and 1.6× higher than that found for *Anabaena variabilis* [30]. Comparing its lipid production with other strains of the *Phormidium* Kützing ex Gomont genus, we obtained 2.1× more lipids than that found in *Phormidium* sp. FW01 and 1,9× more than *Phormidium* sp. FW02 as reported in Yadav et al. [34], and 2.5× more than in *Phormidium valderianum* Gomont [35].

The screening of strains for lipid production assumes a significant role in biotechnology, since the inherent production of lipids in already known cyanobacteria species is relatively low [36] and looking for new promising species can be a step towards a future production of biofuel [35]. We emphasize that *Phormidium* sp. and *S. brasiliense*, in addition to having a high lipid content among the studied species, also showed high growth rate in the CPBR. Based on these results and targeting lipid-rich biomass, we suggest that *Phormidium* sp. together with *S. brasiliense* are promising strains for lipid production. We suggest that the lipid levels obtained here are the result not only of the endogenous characteristics of the strains, but also of the internal adjustments of the CPBR used. We complement that the multiple positive correlations obtained for total lipids with all other biomolecules produced by the studied cyanobacteria, except phycocyanin, are a demonstration that in cyanobacteria other important functions, in addition to reserve, are performed by lipids.

**Fig. 4:**
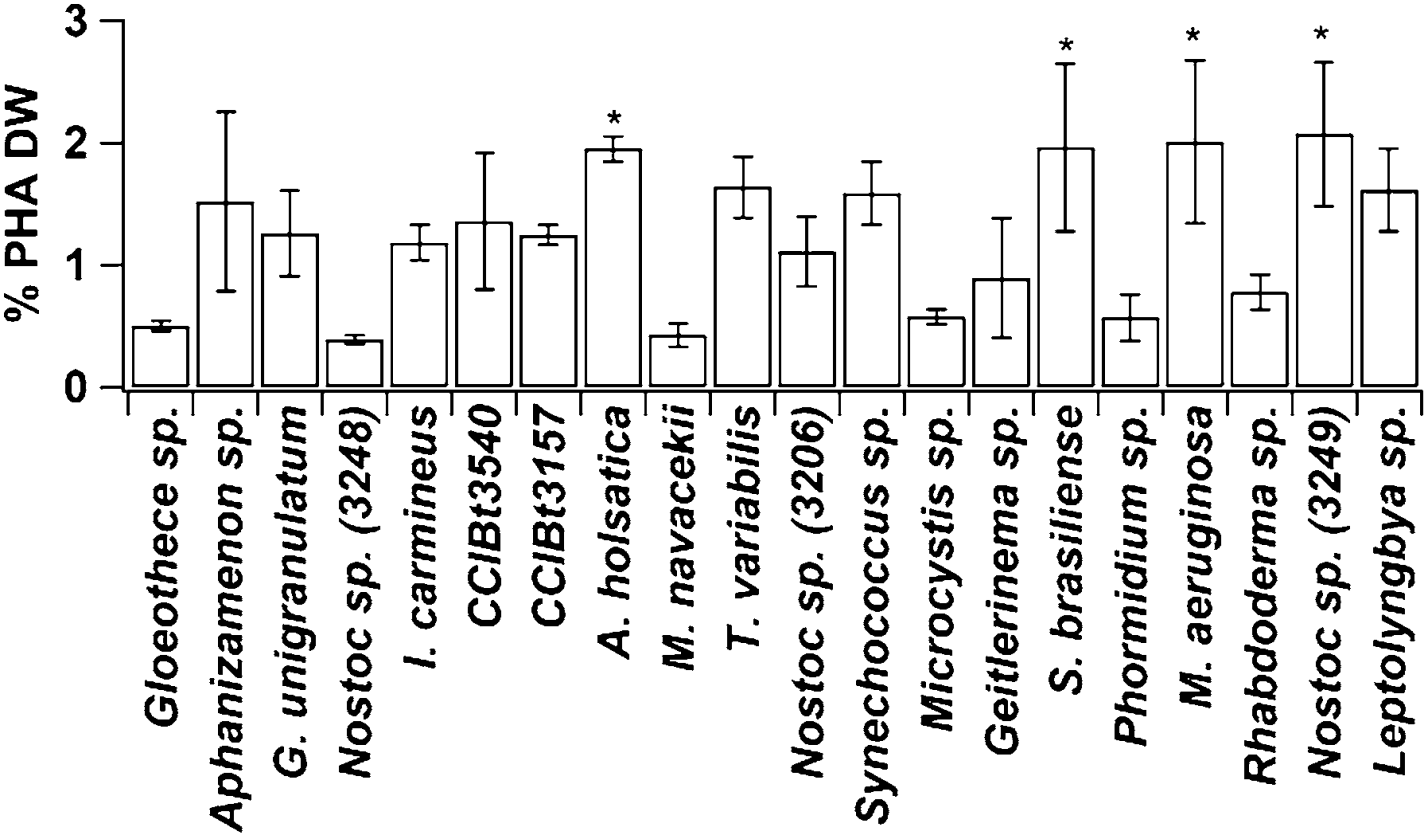
Percent of PHA in the biomass of the 20 strains of cultured cyanobacteria. Error bars represent the standard deviation from the mean (n = 3). The asterisk (*) represents the highest value (ANOVA, p < 0.05)

The percent of PHA in the strains we studied is shown in Fig. 4. PHAs are esters that accumulate intracellularly in cyanobacteria (and bacteria) acting as energy reserve [10]. As a biodegradable polymer [14], there is great interest in strains that produce PHAs [10, 12]. Among the species we investigated there was a variation of 0.4 to 2% of PHA in relation to the dry biomass. According to Sharma et al. [37] who investigated PHAs in more than 100 strains of cyanobacteria, about 70% of them contained PHA in concentrations ranging from 0.04% to 6% of dry biomass. In a study with 23 species of cyanobacteria, 20 had PHA levels ranging from 0.51% (*S. platensis*) to 6.4% (*Nostoc muscorum*) [2]. In our study, *A. holsatica, S. brasiliense*, *M. aeruginosa* and *Nostoc* sp. (CCIBt3249) presented ~ 2% of PHA in the biomass, which was 2 × higher than that found in *A. platensis* [9], and similar to the values obtained in *Synechococcus subsalsus* Skuja and *Spirulina* sp. [38], and *Microcystis aeruginosa* in another study that evaluated 25 species of cyanobacteria [39].

There is a need to screen new species for obtaining, characterizing and processing PHA [2]. Under ideal cultivation conditions, it makes sense that in this study we did not observe positive correlation between PHA and any analyzed parameter. Our PHA results serve as indication for strains that intrinsically produce PHAs under ideal cultivation conditions. Further investigation may look into manipulation strategies to increase accumulated PHAs, such as limiting nutrients. The literature has shown that up to 5 × more PHAs were obtained under stressful growth conditions [6, 12, 14, 40]. It is known that heterotrophic bacteria produce large amounts of the biopolymer [38, 39], but with a high substrate cost [12]. The point of using cyanobacteria against bacteria is that cyanobacteria are photoautotrophic organisms and require minimal nutrients [12].

### Antioxidant activity

**Fig. 5:**
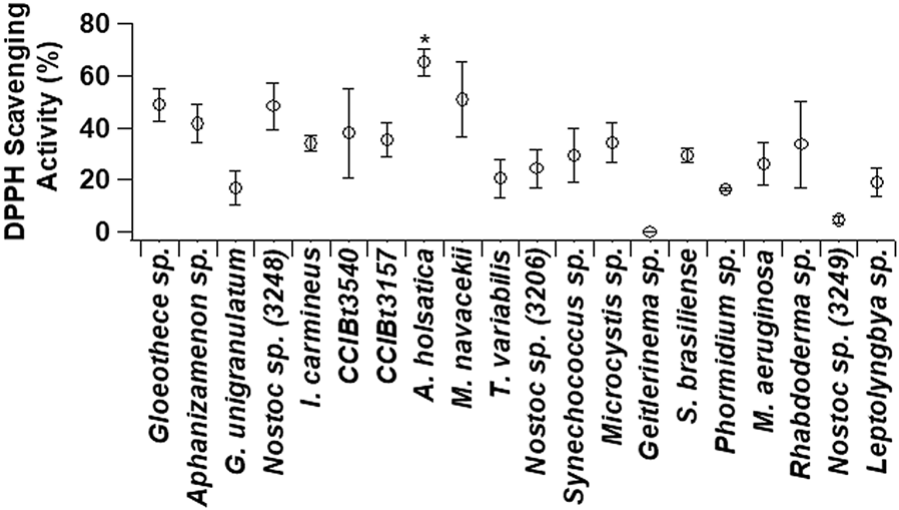
DPPH radical scavenging activity of the 20 strains of cultured cyanobacteria. Error bars represent the standard deviation from the mean (n = 3). The asterisk (*) indicates the highest value (ANOVA, p < 0.05)

The antioxidant activity of extracts obtained from the lyophilized biomass of the species we studied are reported in Fig. 5. In cyanobacteria, antioxidant compounds are responsible for fighting and eliminating free radicals [41], and it can be expected that different strains have different antioxidant activity. In this study, the strains showed variation in antioxidant activity against DPPH radicals. The highest value was found in *A. holsatica* with 65% activity, followed by *M. navacekii* (51%), *Gloeothece* sp. (49%), *Nostoc* sp. (CCIBt3248) (45%) and *Aphanizamenon* sp. (41%).

The activity of *A. holsatica* in our study was similar to that found by Morone et al. [42] in ethanolic extract of *Synechocystis salina* Wisłouch LEGE 06099, which was 60%. Our values for *A. holsatica* are also close to those found for *S. platensis* (68%) in hexane extract [43]. Evaluating antioxidant activity requires considering the solvent in which the extract was produced, as it depends on the solvent. Some differences that we observed from ours against the activity reported in literature may be a result of the different solvents used [44]. In a study comparing the extraction solvents hexane, petroleum ether, ethanol and water, higher antioxidant activities were obtained for *S. platensis* in hexane [43]. Efficient extraction is related to solvent penetration into the cell and dissolution of target compounds related to the combined solvent/antioxidant polarity [45]. In our study, we used methanolic extracts which, according to the recent literature, phenolic compounds [46] and carotenoids [45] are better obtained; both compounds participate in the antioxidant activity [41]. Therefore, the species studied here are a natural source of free radical scavenging compounds, being an alternative to synthetic antioxidants and may be of interest for use in the food industry or similar [43].

## Conclusion

This article evaluated the growth, biochemical composition and photosynthetic parameters of 20 species of cyanobacteria, among them species studied for the first time. We used a photobioreactor that enabled high-biomass production of various cyanobacteria under healthy conditions for 6 days. *Leptolyngbya* sp. and *Nostoc* sp. CCIBt3249 showed higher growth rates, and cultures of *Leptolyngbya* sp., *Nostoc* sp. CCIBt3249, *Phormidium* sp. and *Microcystis* sp. the highest dry biomass (0.8 g L^−1^).

Among the investigated species, the highest concentration of chlorophyll *a* was found in *Leptolyngbya* sp., *Rhabdoderma* sp. and *Phormidium* sp. *Rhabdoderma* sp. stood out in the production of carotenoids (4 µg mL^−1^) and *Leptolyngbya* sp. in phycocyanin (62 µg mL^−1^). The percent composition of proteins, carbohydrates and lipids in the dry biomass remained constant throughout the 144 h of growth. Considering the contents of biomolecules in the cyanobacterial biomass, *G. unigranulatum* and *Gleiterinema sp.* had the highest protein content (75%), *M. navacekii* carbohydrates (30%) and *Phormidium* sp. and *S. brasiliense* of lipids (15%).

The species *A. holsatica, S. brasiliense, M. aeruginosa* and *Nostoc* sp. (CCIBt3249) had the highest PHA content in the biomass (2%) among the studied species. In addition, *A. holsatica* showed high antioxidant activity (65%), being an alternative to synthetic antioxidants.

This study is an important contribution to the biotechnology industry, indicating promising strains for obtaining specific biomolecules.

## Material and methods

The 20 species of cyanobacteria studied were provided by Profª Dr. Célia Leite Sant'anna, from the Institute of Environmental Research, Secretary of the Environment, São Paulo, Brazil. All strains (Table 2) are identified as CCIBt (Culture Collection of the Botanic Institute) and their respective code. The proposed cyanobacteria strains were all isolated from Brazilian environments and chosen because of the lack of information on their physiology. Thus, we included species from different habitats, such as *Leptolyngbya* sp. isolated from the Pantanal (MS, Brazil), a tropical marsh area, and *Gloeothece* sp. from the soil of the Atlantic Jungle (Brazil). In addition, here we include species described for the first time, such as the genus *Inacoccus* described by Gama, J. Rigonato, M. F. Fiore & C. L. Sant’Anna [47], and species with extensive characterization in the literature, such as those of the genus *Nostoc*.

**Table 2:** Species of cyanobacteria investigated in the prospection

Species	Strain code	Growing medium	Ik µmol fótons m^−2^ s^−1^
*Gloeothece* sp. (C.Nägeli)	CCIBt3513	ASM-1	57 (4.26)
*Aphanizamenon* sp. (Morren ex Bornet & Flahault)	CCIBt3148	ASM-1	293 (5.34)
*Geitlerinema unigranulatum* (J.Komárek & M.T.P.Azevedo)	CCIBt3231	BG11	148 (26.62)
*Nostoc* sp. (Vaucher ex Bornet & Flahault)	CCIBt3248	BG11	145 (2.46)
*Inacoccus carmineus (*W.A.Gama, J.Rigonata, M.F.Fiore & C.L.Sant'Anna)	CCIBt3411	ASM-1	196 (9.12)
*Não identificado*	CCIBt3540	ASM-1	79 (3.05)
*Não identificado*	CCIBt3157	BG11	241 (4.32)
*Aphanocapsa holsatica* (G.Cronberg & Komárek)	CCIBt3053	BG11	247 (4.30)
*Microcystis novacekii* (Komárek Compère)	CCIBt3189	ASM-1	399 (5.48)
*Trichormus variabilis* (Komárek & Anagnostidis)	CCIBt3122	BG11	227 (2.77)
*Nostoc* sp. (Vaucher ex Bornet & Flahault)	CCIBt3206	BG11	146 (0.55)
*Synechococcus* sp. (Nägeli)	CCIBt3050	BG11	238 (10.41)
*Microcystis* sp. (Kützing)	CCIBt3078	ASM-1	283 (2.56)
*Geitlerinema* sp. (Anagnostidis)	CCIBt3241	BG11	259 (1.80)
*Sphaerocavum brasiliense* (De Azevedo & C.L.Sant' Anna)	CCIBt3179	BG11	340 (4.63)
*Phormidium* sp. (Kützing ex Gomont)	CCIBt3280	BG11	242 (15.47)
*Microcystis aeruginosa* (Kützing)	CCIBt3174	BG11	135 (1.72)
*Rhabdoderma* sp. (Schmidle & Lauterborn)	CCIBt3165	BG11	275 (0.99)
*Nostoc* sp. (Vaucher ex Bornet & Flahault)	CCIBt3249	BG11	187 (4.91)
*Leptolyngbya* sp. (Anagnostidis & Komárek)	CCIBt1046	BG11	117 (16.21)

Each species has its own identification and respective saturating irradiance values (Ik). Numbers in parentheses correspond to the standard deviation of the mean (n = 3)

Two types of culture medium were used, namely BG11 [48] and ASM-1 [49], referring to the culture medium in which they were provided (Table 2). The culture media were sterilized in an autoclave (20 min, 121 °C, 1 bar; AV Phoenix Luferco, Brazil) and, for all cultures the initial inoculum was obtained from exponentially growing cells with ~ 0.05 µg mL^−1^ of chlorophyll *a *in vivo determined in a fluorometer (Turner Designs, Trilogy, USA). The total duration of the cultures was 6 days (144 h).

The cyanobacteria were grown under controlled temperature (24 ± 1 °C), pH, light intensity, and CO_2_ concentration. Cultivations (2L) were carried out in a tubular borosilicate glass photobioreactor (CPBR) with 3L capacity. Illumination was performed from inside the CPBR by light emitting diodes (LED). The photosynthetic active radiation (PAR) was determined daily in the CPBR and kept constant throughout the cultivation; the photoperiod was 12 h light:12 h dark. Each species was cultivated at its respective saturating irradiance (Table 2), previously determined from rapid light curves using a PhytoPAM II equipment (Heinz Walz, Germany). The cultivation was mixed by air bubbling using commercial pumps (Big Air, AZ30) with porous stones at the end of the pipe. The initial pH of the medium was adjusted to 7.4. After the start of cultivation, the pH was kept adjusted by the automatic insertion of CO_2_ (25% v/v) in a mixture with argon (75% v/v). The CO_2_ insertion system was activated when the culture pH reached 8.4 and turned off at pH 7.8.

### Growth parameters

Daily, 3 mL of culture were sampled to monitor absorbance at 684 nm and 570 nm, referring to peak absorbance of chlorophyll *a* and particulate matter, respectively. Measurements were performed in a spectrophotometer (NANOCOLOR, Macherey–Nagel, Germany). Daily, 3 mL of culture were also collected to determine the concentration of chlorophyll *a *in vivo (Ca). In vivo Ca was obtained from fluorescence measurements (in relative fluorescence units, RFU) using a digital fluorometer (Turner Designs, Trilogy, USA). The calculation of the pigment concentration was based in a calibration curve made with chlorophyll *a* extract obtained from a culture of *Chlorella sorokiniana* and plotted against RFU from the Turner fluorometer and based in a calibration curve. The standard calibration curve of the in vivo chlorophyll a concentration was y = 11369x – 8.0744 and R^2^ = 0.9769.

Growth rate (µ, d^−1^) was calculated from growth curves based on the natural logarithm of chlorophyll *a *in vivo (µg mL^−1^) *versus* culture time. The curve was fitted using linear regression and its slope is equivalent to µ.

### Biochemical composition

The biochemical composition was determined at 48 h (exponential growth phase) and 144 h of cultivation (beginning of stationary growth phase). Samples were centrifuged in a refrigerated centrifuge (Thermo Scientific, Sorvall Legend XTR, USA) for 20 min at 4000 rpm. For proteins and carbohydrates, 10 and 30 mL were collected, respectively. Then, their respective pellets were frozen (− 80 °C) until further analysis. The determination of total proteins followed the protocol by Slocombe et al. [50], with extraction using hot trichloroacetic acid and alkaline solution, and bovine albumin (2 mg mL^−1^) as standard solution. Total carbohydrates were determined as described in Albalasmeh et al. [51] with glucose as standard. For phycocyanin 10 mL of culture was centrifuged and the pigment determined according to Yéprémian et al. [52]. Chlorophyll *a* and carotenoids were quantified by filtering 3 mL of culture through a cellulose acetate filter (Unifil, Brazil, 0.22 µm) and determined as proposed in Wellburn [53]. The total lipid content was determined gravimetrically by filtering 100 mL of culture through preciously baked (muffle furnace, 400 °C, 8 h) glass fiber filters (Sartorius—Germany). Total lipids extraction was done with chloroform and methanol and the extract was dried before the gravimetric quantification on a microanalytical balance with 1 µg precision (Mettler Toledo, XPE26). Finally, for the determination of biomass dry weight (DW), a volume of 10 mL was sampled at 48 h and 144 h and filtered through cellulose acetate filters (Unifil, Brazil, 0.22 µm) previously dried at 100 °C in an oven (Qualxtron, Brazil), and the mass measured in a 1 µg precision balance (Mettler Toledo, XPE26). Biomolecules are reported as per unit dry biomass weight.

The DPPH antioxidant activity was determined in lyophilized biomass with 144 h of experimental time. Extraction was performed following the method described in Pires et al. [54]. For each sample, three extraction cycles were performed with 1 mL of methanol each, vortexed with beads and subsequent centrifugation (Legend XTR; Thermo Scientific, USA). The PHA content was determined in lyophilized biomass obtained on the last day of cultivation. Extraction and quantification followed the method proposed by Costa et al. [11], with cell disruption with sodium hypochlorite (4%), extraction in hot chloroform, precipitation in methanol and evaporation of the solvent in an oven (12 h) at controlled temperature (30 °C). The dried extract was quantified gravimetrically using a microanalytical balance with 1 µg precision (Mettler Toledo, XPE26). The accumulation of PHAs was calculated according to Eq. 1.

1 $$\mathrm{Y}=(mp \times 100) / mb$$

where Y is the accumulation of PHAs expressed in percentage, *mp* is the mass of PHAs obtained in grams and *mb* is the mass of the biomass used in the extraction and expressed in grams.

### Statistical analysis and graphics

Statistics were performed using the MiniTab17 software. Results were analyzed using one-way analysis of variance (ANOVA) and Tukey's test with 95% confidence to detect significant differences among species. Graphs were made using the IgorPro 6.0.5 program (WaveMetrics, USA).

## Supplementary Information

**Additional file 1: Figure S1.** Total biomolecules (µg mL^−1^) in the 20 species of cultured cyanobacteria. In (a) determination in 48 h and in (b) 144 h of experimental time. White bars represent lipids, black bars carbohydrates and gray bars proteins. Error bars represent the standard deviation from the mean (n = 3). The asterisk (*) indicates the highest value (ANOVA, p < 0.05).

## Data Availability

Datasets used and/or analyzed during the current study are available from the corresponding author upon reasonable request.
