# Supplementary figures and images for: Study of the growth and biochemical composition of 20 species of cyanobacteria cultured in cylindrical photobioreactors
Source: Microb Cell Fact. 2023 Feb 24;22:36. doi: 10.1186/s12934-023-02035-z (PMC9951496; doi:10.1186/s12934-023-02035-z)

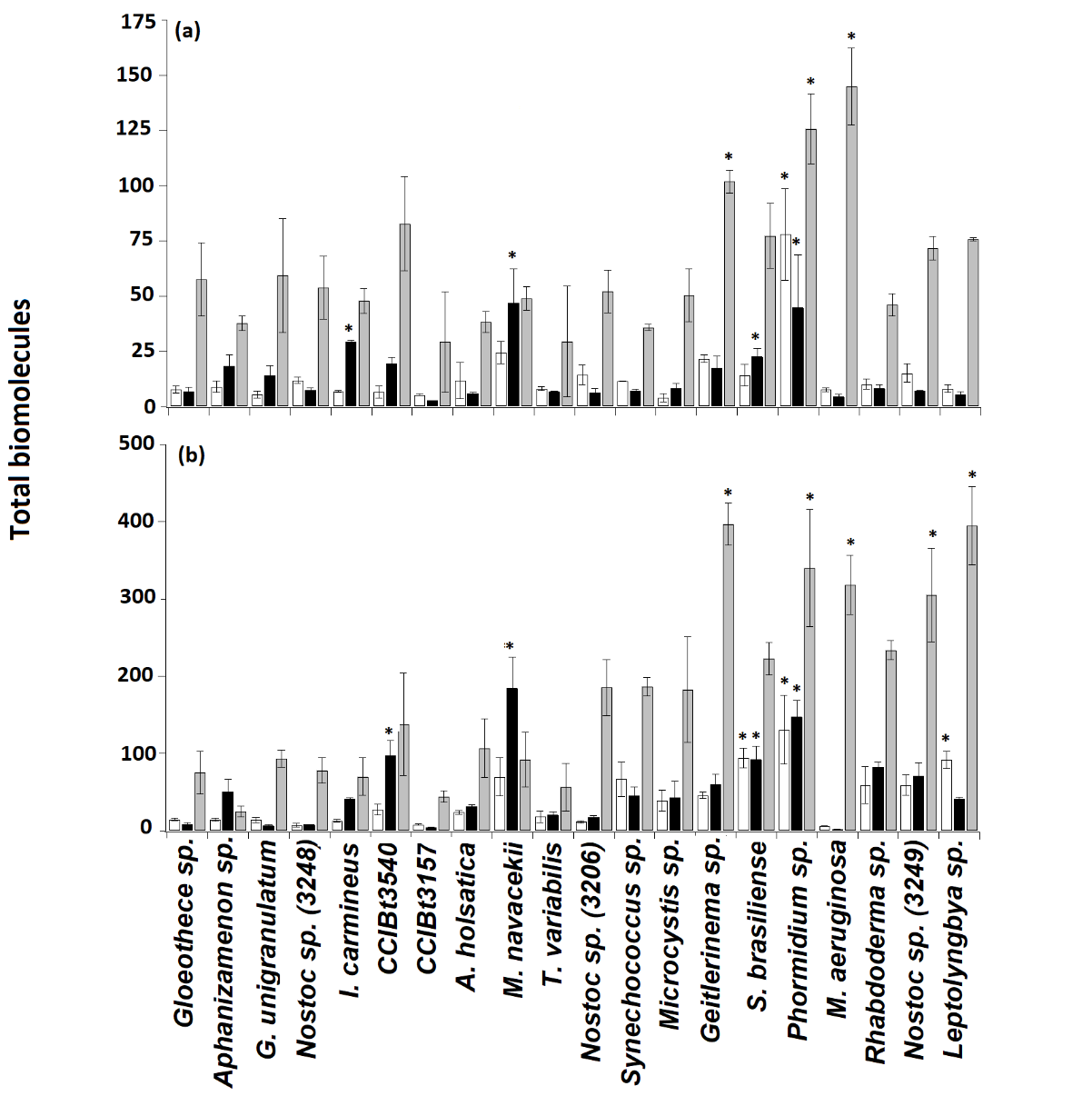

Supplement: Supplementary file 1 — Additional file 1: Figure S1. Total biomolecules (µg mL−1) in the 20 species of cultured cyanobacteria. In (a) determination in 48 h and in (b) 144 h of experimental time. White bars represent lipids, black bars carbohydrates and gray bars proteins. Error bars represent the standard deviation from the mean (n = 3). The asterisk (*) indicates the highest value (ANOVA, p < 0.05). [file 12934_2023_2035_MOESM1_ESM.tif]
